# Analysis of the Impacts on the Psychological Changes of Chinese Returning College Students After the Outbreak of the 2019 Coronavirus Disease

**DOI:** 10.3389/fpubh.2022.916407

**Published:** 2022-05-25

**Authors:** Yingying Xue, Kwak Han Pyong, Sae Sook Oh, Yingying Tao, Taofeng Liu

**Affiliations:** ^1^Institute of Physical Education, Yangzhou University, Yangzhou, China; ^2^Sports Science Department, Kyonggi University, Suwon, South Korea; ^3^Department of College Physical Education, Communication University of Zhejiang, Hangzhou, China; ^4^Zhengzhou University Physical Education Institute (Main Campus), Zhengzhou, China; ^5^Department of Physical Education, Sangmyung University, Seoul, South Korea

**Keywords:** COVID-19, college students, alienation, school happiness, psychological changes

## Abstract

This work aims to analyze the impacts on the psychological changes of Chinese returning college students after the outbreak of the 2019 coronavirus disease (COVID-19). A questionnaire survey is used to take 1,482 college students who returned to school after the epidemic as the research objects. The Chinese college students' knowledge of the epidemic, alienation in physical education class, school happiness, and expectations for a healthy life in the future are investigated and analyzed. The research results manifest that Chinese returning college students have relatively poor awareness of COVID-19, and the overall degree of alienation in physical education classes after the epidemic is low, with an average score of 3.55 ± 1.018. The overall level of school happiness is high, with an average score of 4.94 ± 0.883; the overall level of expectation for a healthy life in the future is high, with an average score of 3.50 ± 0.840. It denotes that the epidemic has a great psychological impact on returning college students, and it is necessary to strengthen mental health education for college students after COVID-19. It provides a sustainable theoretical reference for the formulation of psychological intervention measures for returning college students.

## Introduction

A major infectious disease broke out in China at the end of 2019, that is, pneumonia caused by a new type of coronavirus, which is called “Corona Virus Disease 2019” by the World Health Organization (WHO) (1, 2). The outbreak of Corona Virus Disease 2019 occurred in December 2019, mainly in Wuhan City, Hubei Province, China. The cause of the disease was unknown. Epidemiological investigations found that they had all been to the Huanan Seafood Market, and were finally diagnosed as acute respiratory disease caused by new coronavirus infection (3). Comrade Tedros of the WHO announced in Geneva, Switzerland on February 11, 2020, that the disease was named as “COVID-19” (4). Meanwhile, the National Health Commission (NHC) issued a notice on February 22 to change its English name to “COVID-19.” After that, WHO declared the current COVID-19 outbreak a global pandemic on March 11 (5, 6). COVID-19 is an infectious disease of the respiratory tract caused by coronavirus type 2 infection, which can induce severe acute respiratory syndrome (7). Since the onset of the disease, all provinces, municipalities, and autonomous regions in China have taken strict prevention and control measures to avoid travel and crowd gathering as much as possible, and have achieved good results. According to the existing case data, the main manifestations of COVID-19 include fever, dry cough, and fatigue. A small number of patients will have symptoms of the upper respiratory and digestive systems, such as nasal congestion, runny nose, and diarrhea. Severely ill patients usually suffer symptoms of dyspnea after 7 days of onset, can be complicated by Acute Respiratory Distress Syndrome (ARDS) in a short period of time, and then develop septic shock and metabolic acidosis (refractory), multiple organ failure, etc. (8–10). COVID-19 is a Class B infectious disease managed at the national level, and a public health emergency. Since the establishment of the People's Republic of China, it has spread the fastest, and it is the most widespread and the most difficult to prevent and control infection among major public health events (11). College students are in an active period of physical and mental development. Faced with the dual pressures of the epidemic and their studies, college students are more likely to have negative emotions, develop bad living and exercise habits, and decline in physical fitness.

So far, measures for the prevention and control of the COVID-19 epidemic taken by Chinese government have developed from the initial emergency state to the current normalization work. Full-scale implementation began, and in the meantime, the resumption of normal work and production has been actively underway (12). The Guidelines for the Prevention and Control of Novel Coronavirus Pneumonia in Colleges and Universities proposed in March 2020 stated that the huge pressure caused by the new coronary pneumonia can easily induce psychological stress such as anxiety, depression, and hypochondriasis. During the period of the epidemic, students were isolated and managed at home. The government and schools encouraged and supervised students to exercise through online physical education teaching and assignment of exercise homework. However, home exercise mainly relies on students' conscious self-exercise, and schools lack effective monitoring methods (13, 14). This has a great impact on college students' self-efficacy, life behavior, mental health, learning adaptability, social responsibility and values (15, 16). With the continuous progress and development of information technology, the research methods in psychology have also undergone great changes since the 1940s (17). In the 1970s, some experts tried to use computers to carry out experiments in psychology, which led to the first revolution in the psychology. In the 1990s, the birth of the online psychological questionnaire was the second revolution in psychology due to the advancement of network technology. At this stage, experts and scholars in the psychology established virtual psychological laboratories on the Internet. The advent of the era of big data has prompted the beginning of the third revolution in psychology. The samples of big data are featured with large amount of data, various types, fast traffic, and great potential significance. They have gradually become valuable resources for researchers to observe the characteristics and laws of human psychological behaviors (18–21).

Compared with previous studies, psychological research based on big data technology has great advantages. This shows that psychological research in the past has focused more on the explanation of a certain psychological response. Big data technology can predict and intervene certain psychological reactions on the basis of interpretation (22). Furthermore, the research method based on big data technology can conduct an overall analysis of all data, avoid the sampling error of previous research methods from the sample to the overall promotion, reduce the experimental error caused by improper control of experimental conditions, and greatly improve the efficiency and scale of data processing. In terms of time validity, real-time collection and analysis of psychological states avoid the deviation of analysis results caused by the lag of data collection in traditional research methods (23, 24). The innovation is the use of regression analysis to examine the status of COVID-19-related knowledge among Chinese returning college students after the COVID-19 pandemic, as well as changes in their alienation in physical education classes, school happiness, and expectations for a healthy life in the future. This study aims to provide a sustainable theoretical reference for the formulation of psychological intervention measures for returning college students. The research framework is to first introduce the research objects, observation indicators and survey methods of the questionnaire; then regression analysis is used to analyze the changes in Chinese returning college students' alienation in physical education classes, school happiness, and expectations for a healthy life in the future after the COVID-19 pandemic.

## Materials and Methods

### Research Objects

The random sampling is adopted to investigate the college students who returned to school after the COVID-19 pandemic in different provinces and cities in China. From June to October 2020, a questionnaire survey was conducted on 1,482 college students who were approved to return to school under the principle of informed consent. The included college students have to meet the following criteria: full-time college students returning to school after the epidemic (undergraduate, master, and above) and returning college students who are informed with this experiment and cooperate with the investigation. If any student meets the below items, it has to be excluded: students who have left or dropped out of school; and students who are on-the-job, self-taught adults, or receiving correspondence education. All respondents have fully understood the situation and signed the informed consent forms, and the Medical Ethics Committee of our hospital has also been informed and agreed to implement it.

### Observation Indicators

The general information of included students is investigated, including gender, age, grade, major, and place of origin. Cognition of COVID-19 includes what type of infectious disease is the COVID-19 and its route of transmission, the main symptoms, the method of transmission, and the personal preventive measures. The situation related to the COVID-19 epidemic includes what information about the epidemic situation is the most concerned about, how much time it takes to pay attention to the epidemic situation every day, how to obtain relevant information about the epidemic situation, and the attention in information about the epidemic situation reported by the media. College student alienation scale (25): there are 52 questions in total, the higher the score, the stronger the alienation. The school happiness scale for college students (26): there are 36 questions, the higher the score, the higher the level of happiness. The expected measure of future healthy life (27): there are 16 questions in total, the higher the score, the higher the expectation.

### Investigation Methods

The questionnaire survey is carried out by scanning the questionnaire star QR code with a smartphone. After the consents of the participants are obtained, the mobile phone WeChat is adopted to scan the code to open the general information about returning college students after the COVID-19 pandemic, COVID-19 awareness, epidemic-related situations, alienation in physical education classes, school happiness, and future life expectations. The researcher writes the instruction language uniformly, follows the principle of free and informed consent, and explains the purpose of the questionnaire survey and the confidentiality principle in detail to the participants. In the context of the questionnaire star operation, effective permission settings are carried out, that is, an IP address can only answer the questionnaire online once, and the filling time is not <5 min.

### Linear Regression Analysis

Linear regression refers to a regression analysis that simulates the relationship between one or more independent variables and dependent variables through the least squares function obtained from the linear regression equation (28). This least squares function is a linear combination of model parameters and one or more regression coefficients. There are also such relationships in our daily life, such as the irrelevant relationship between random numbers, the uncertain relationship between individual height/weight, and the definite relationship between the area/radius of a circle. Regression analysis is one of the mathematical methods that is often used to study the existence or absence of correlations between variables that are not completely determined. When only one independent variable is used for regression analysis, it is called univariate linear regression analysis; when there are multiple variables, it is called multiple linear regression analysis.

I. Univariate linear regression analysis

Univariate linear regression analysis, also known as Simple Linear Regression (SLR), is the simplest regression analysis, and there are regression models that are widely used in various fields of life. Its regression equation is given as follows:


(1) $$\begin{array}{r} A\text{=}\alpha +\beta B+\varepsilon \end{array}$$

In general, the least squares method is used to evaluate the minimum errors α and β from the sample (*a*_*i*_, *b*_*i*_) (i = 1,2,…,*n*), ε represents the residual, and the objective is to minimize the residual sum of squares:


(2) $$\begin{array}{r} \overset{n}{\underset{i=1}{\sum }}\varepsilon_{i}^{2}=\overset{n}{\underset{i=1}{\sum }}{\left(a_{i}-\alpha -\beta b_{i}\right)}^{2} \end{array}$$

The extreme value can be obtained by using the differential method. In equation (2), the first-order partial differential is made with α and β, respectively, and it is equal to 0 to obtain $\overset{⌢}{\alpha }$ and $\overset{⌢}{\beta }$:


(3) $$\begin{array}{r} \overset{⌢}{\alpha }=ā-\bar{b}\overset{⌢}{\beta } \end{array}$$

(4) $$\begin{array}{r} \overset{⌢}{\beta }=\frac{\overset{n}{\underset{i=1}{\sum }}\left(b_{i}-\bar{b}\right)\left(a_{i}-ā\right)}{\overset{n}{\underset{i=1}{\sum }}{\left(b_{i}-\bar{b}\right)}^{2}} \end{array}$$

II. Multiple linear regression analysis

The equation for the multiple linear regression prediction model is expressed as follows:


(5) $$\begin{array}{r} {\overset{⌢}{A}}_{t}=\alpha +\beta_{1}b_{1}+\beta_{2}b_{2}+\beta_{3}b_{3}+...+\beta_{n}b_{n} \end{array}$$

b_1_-b_n_ represent independent variables; α, β_1_-β_n_ express the parameters of the linear regression equation. The binary linear regression analysis model with only two independent variables is the general form of the multiple linear regression model, which is a method for analyzing the correlation between two independent variables and one dependent variable. Its regression equation is given in equation below:


(6) $$\begin{array}{c} {\overset{⌢}{A}}_{t}=\alpha +\beta_{1}b_{1}+\beta_{2}b_{2} \end{array}$$

In the above equation, ${\overset{⌢}{A}}_{t}$ represents the dependent variable, b_1_ and b_2_ represents the independent variables; α, β_1_, and β_2_ indicate the parameters of the linear regression equation. α, β1, and β2 can be obtained by solving below equation:


(7) $$\{\begin{array}{l} \sum a=n\alpha +\beta_{1}\sum b_{1}+\beta_{2}\sum b_{2}\\ \sum b_{1}a=\alpha \sum b_{1}+\beta_{1}\sum b_{1}^{2}+\beta_{2}\sum b_{1}b_{2}\\ \sum b_{2}a=\alpha \sum b_{2}+\beta_{1}\sum b_{1}b_{2}+\beta_{2}\sum b_{2}^{2} \end{array}$$

### Ensemble Learning Method

There is a famous theory of “no free lunch” in machine learning, which states that no algorithm can maintain its accuracy all the time. Therefore, the concept of ensemble learning appears (29). The main idea of ensemble learning is to use a certain strategy to aggregate multiple base classifiers to obtain higher accuracy. Different base classifiers can use different algorithms, parameters, features, training data sets, etc. The basic idea of Bagging, Boosting, and Stacking is to integrate three strategies. Among them, the basic idea of the Stacking strategy (30) is to train a variety of base classifiers {*D*_1_, *D*_2_, ..., *D*_*N*_}. In addition, the Stacking strategy uses the output results {*q*_*i*1_, *q*_*i*2_, ..., *q*_*iN*_, *a*_*i*_} of these base classifiers as input data, retrains to obtain a new model, and obtains the final result. output result.

Boosting tree is a boosting method based on the decision tree function. When it faces the classification task, this decision tree represents a binary decision tree; In the regression problem, the decision tree represents the regression tree, and the boosting tree can be regarded as an additive model of the decision tree:


(8) $$\begin{array}{r} f_{N}\left(b\right)=\overset{N}{\underset{n=1}{\sum }}T\left(b;{\text{Θ}}_{n}\right) \end{array}$$

In equation (8), Θ_*n*_ refers to the model parameters, *T*(*b*; Θ_*n*_) denotes the decision tree model, and N is the number of trees.

When binary classification is required, boosting tree is a special case of binary decision trees specified by the base classifier of the AdaBoost algorithm.

Gradient Boosting Decision Tree (GBDT) is an optimization that can be used to solve the optimization problem when the loss function of the boosting tree is a general function (i.e., a non-square loss function or a non-exponential loss function). The GBDT algorithm flow is described as follows. The value of the negative gradient of the loss function in this model plays a key role in the gradient boosting algorithm, which is an algorithm that is very similar to the steepest descent method:


(9) $$\begin{array}{r} -{\left[\frac{\partial L\left(a_{i},F\left(b_{i}\right)\right)}{\partial F\left(b_{i}\right)}\right]}_{F\left(b\right)=F_{k-1}\left(b\right)} \end{array}$$

When faced with a binary classification, the loss function that can be defined is as follows:


(10) $$\begin{array}{r} L\left(a,F\left(b\right)\right)=\log\left(1+\exp\left(-2aF\right)\right),a\in \left\{+1,-1\right\} \end{array}$$

The process of GBDT is as follows:

Input: training dataset *D* = {(*b*_1_, *a*_1_), (*b*_2_, *a*_2_), ..., (*b*_*N*_, *a*_*N*_)}, a function that is capable of micro-loss is *L*(*a, F*(*b*)), and the number of iterations is K.

Output: the GBDT model *F*_*K*_(*b*).

The model is initialized as follows:


(11) $$\begin{array}{r} F_{0}\left(b\right)=\arg_{\gamma }\min\overset{n}{\underset{i=1}{\sum }}L\left(a_{i},\gamma \right) \end{array}$$

k = 1,2,…,K is cycled:

(a) The desired pseudo-residuals are calculated with equation (12) below:


(12) $$\begin{array}{r} r_{ik}=-{\left[\frac{\partial L\left(a_{i},F\left(b_{i}\right)\right)}{\partial F\left(b_{i}\right)}\right]}_{F\left(b\right)=F_{k-1}\left(b\right)},fori=1,...,n. \end{array}$$

(b) The pseudo-residual is fitted to the base classifier *g*_*k*_(*b*), that is, the training dataset ${\left\{\left(b_{i},r_{ik}\right)\right\}}_{i=1}^{n}$ is adopted to train the base classifier.

(c) The multiplier γ_*k*_ can be calculated by solving a one-dimensional optimization:


(13) $$\begin{array}{r} \gamma_{k}=\arg_{\gamma }\min\overset{n}{\underset{i=1}{\sum }}L\left(a_{i},F_{k-1}\left(b_{i}\right)+\gamma g_{k}\left(b_{i}\right)\right) \end{array}$$

(d) The model is updated:


(14) $$\begin{array}{r} F_{k}\left(b\right)=F_{k-1}\left(b\right)+\gamma_{k}g_{k}\left(b\right) \end{array}$$

### Statistical Analysis

The SPSS 19.0 statistical analysis software package is adopted to enter and analyze the data in this experiment, *t*-test or variance analysis is applied to judge the differences between variables, and the multiple linear regression is selected to analyze the changes on dimensions of alienation and school happiness, expectations for a healthy life in the future. When *P* < 0.05, the difference is statistically significant.

## Results

### General Data

**Figure 1:**
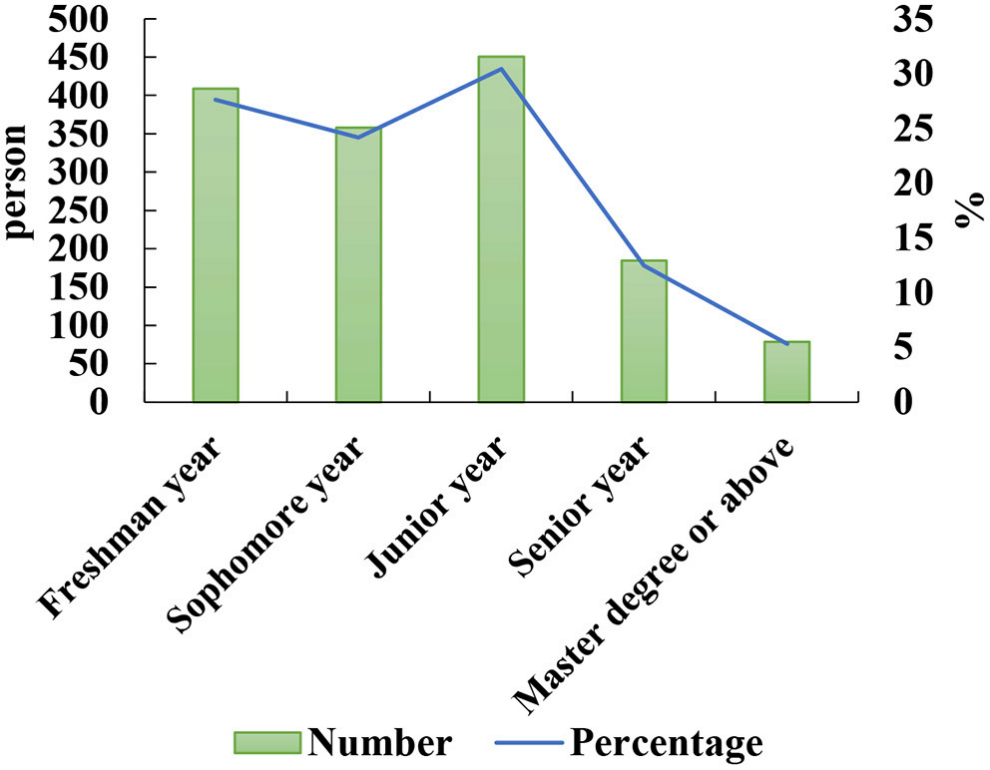
General information on the grades of returning college students.

**Figure 2:**
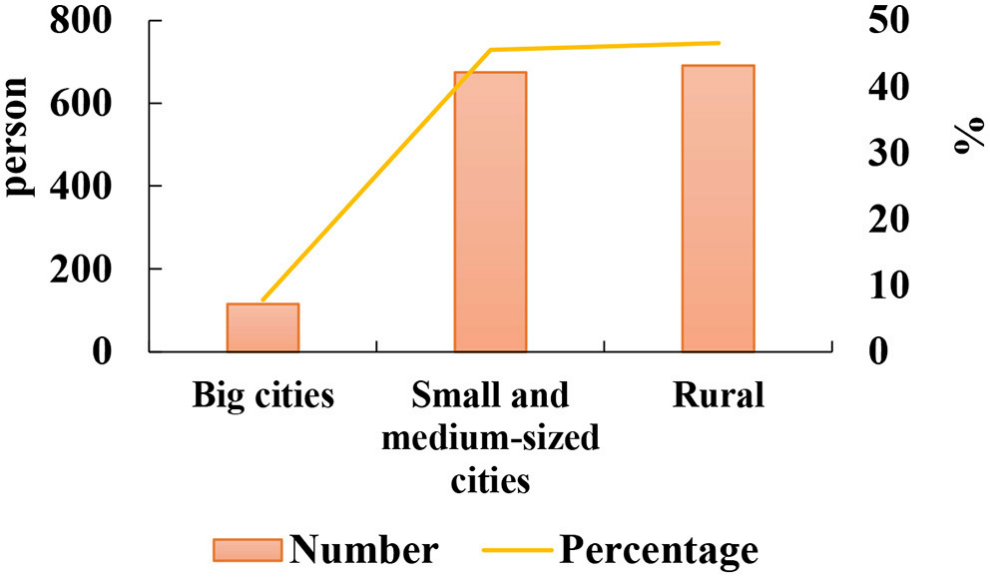
General information on the origin of returning college students.

After the COVID-19 pandemic, the general information of Chinese returning college students (*n* = 1,482) is collected, as shown in Figures 1, 2.

Among them, the numbers of males and females are 519 and 963, respectively, aged between 18 and 30 years old, with liberal arts students accounting for 41.26% and science students accounting for 58.74%. In Figures 1, 2, the proportion of freshman students is 27.6%, junior students is 30.43%, and graduate students and above is 5.33%. Among returning college students, 7.83% came from big cities, 45.55% from small and medium cities, and 46.62% from rural areas.

### Cognition of COVID-19

**Figure 3:**
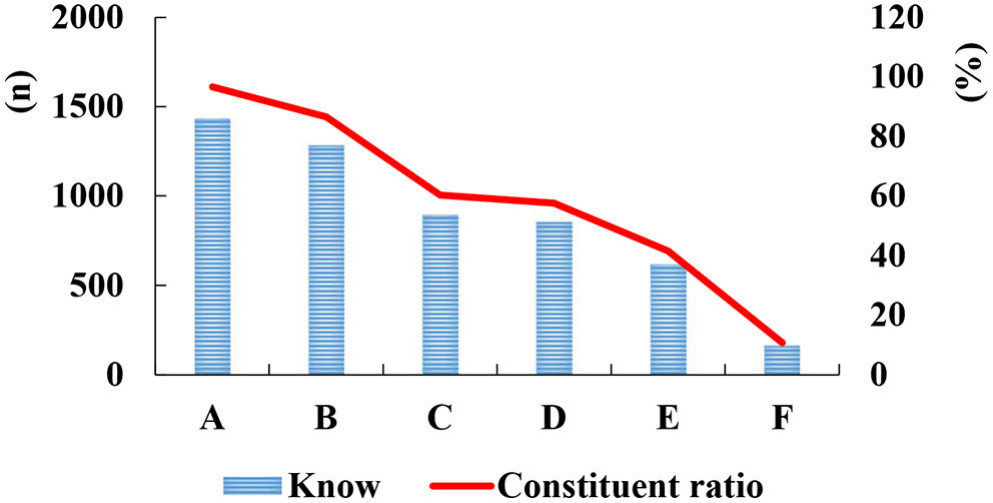
The cognition of Chinese returning college students on the disease after the COVID-19 pandemic.

After the COVID-19 pandemic, we investigated Chinese returning college students' cognition of the disease and found that 96.7% knew that during the COVID-19 epidemic, the protective measures that people need to do include 6 aspects; and only 10.78% knew that the main manifestations of COVID-19 infection included 3 symptoms. Figure 3 showed the details.

In the Figure 3 above, A represents the protective measures that people need to do during the COVID-19 epidemic: washing hands frequently, refusing to eat wild animals, wearing disposable medical masks when entering and leaving public places, trying to avoid entering and leaving crowded places, staying within one meter of people around, opening the window regularly to ventilate, and reduce going out. B represents the main mode of transmission of COVID-19: close contact between people and respiratory droplet transmission. C stands for low case fatality rate of COVID-19; D represents the category B infectious disease of COVID-19 according to the prevention and control management of the category A infectious disease; E represents that if you know that in the absence of effective protective measures, if you have face-to-face communication with infected people, share meals, take public transportation, eat wild animals, or accept express delivery from areas with high epidemic incidence, it will increase the risk of epidemic transmission. F stands for the main clinical manifestations of COVID-19 infection, including fever, dry cough, and fatigue.

### Situation of COVID-19

**Figure 4:**
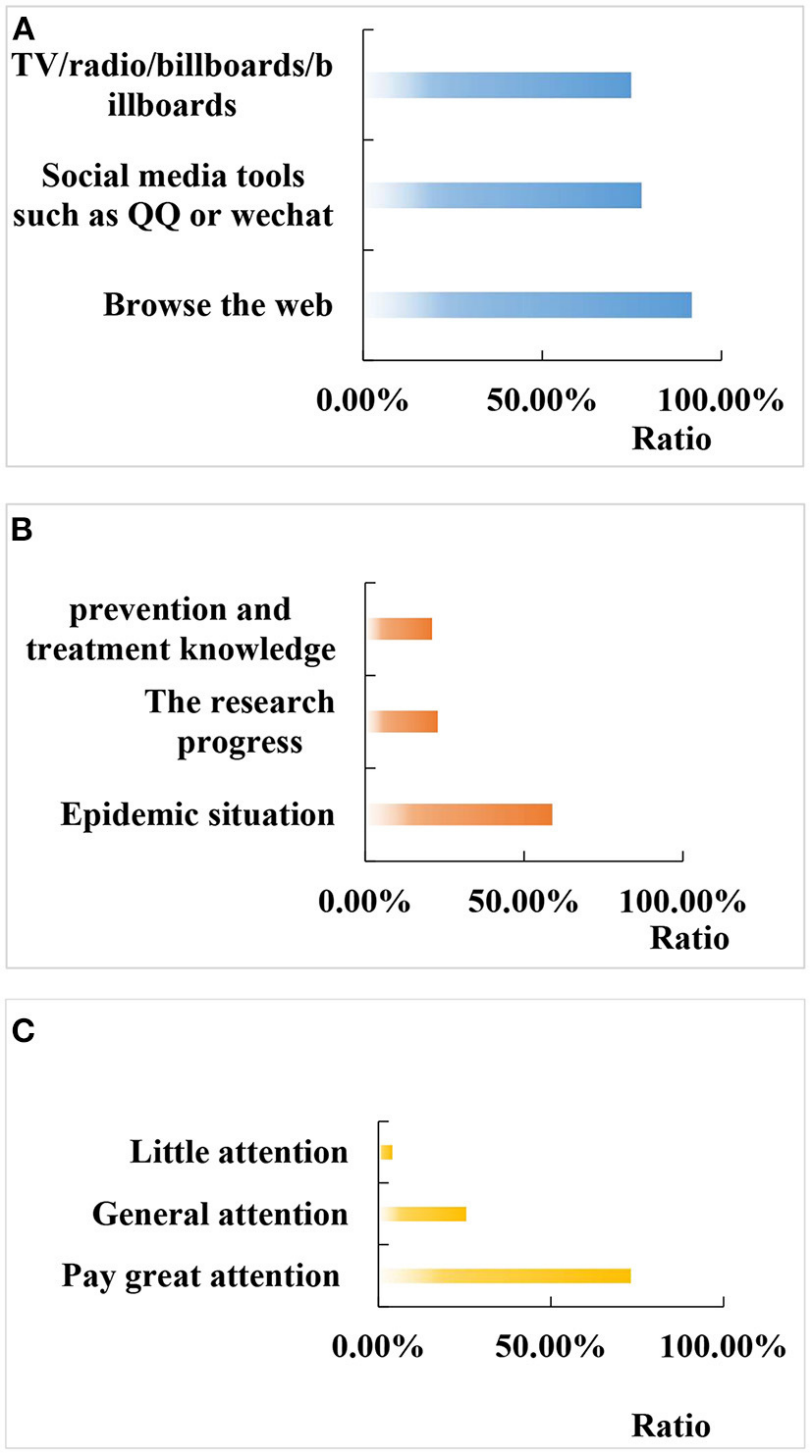
The situation related to the COVID-19. **(A)** Main access way; **(B)** most concerned epidemic information; **(C)** attention to the information related to the epidemic situation reported by the media.

After the COVID-19 pandemic, we investigated the three main ways that Chinese returning college students obtain information related to COVID-19: web browsing (91.70%, 1,359/1,482), social tools such as QQ or WeChat (77.68%, 1,151/1,482), television / radio / billboard / billboard (74.8%, 1,108/1,482). The average time spent paying attention to the epidemic every day is 24.50 ± 22.6 min. The most concerned epidemic information includes: epidemic situation (58.86%, 872/1,482), research progress (22.87%, 339/1,482), and prevention and control knowledge (21.04%, 312/1,482). Concern on media reports on epidemic-related information is explained as: very concerned (73.14%, 1,084/1,482), generally concerned (25.48%, 378/1,482), and rarely concerned (4.05%, 600/1,482). The details are given in Figure 4.

In the above figure, Figure 4A shows the three main ways Chinese returning college students obtain information about COVID-19; Figure 4B shows the epidemic information they are most concerned about; and Figure 4C shows their attention to media reports on epidemic-related information.

### Differences in Alienation and School Happiness in Demographic Data

After the COVID-19 pandemic, the alienation in physical education class and school happiness of Chinese returning college students are compared in the term of demographic data. The results are shown in Table 1. It demonstrates that that among returning college students, the average alienation in physical education class is higher for boys than for girls. The average of freshmen is lower than that of other grades, the average of non-only children is higher than that of only-children, and the average score of students living in rural areas is higher than in other places. In terms of school happiness, among returning college students, the score of upper class students is higher compared with that of lower class students, and the score of students living in urban areas is slightly higher in contrast to that in rural and urban areas.

**Table 1:** Comparison of alienation and school happiness in demographic data.

**Alienation and school happiness**	**Difference in demographic data**
Alienation	The score of boys (4.52 points in average) is higher than that of girls (4.31 points).
	The average score of Freshmen (3.98 points) is lower than the score of other grades (4.39 points).
	The average score of non-only children (4.45 points) is higher than that of only children (4.26 points).
	The average score of students living in rural areas (4.38 points) is higher than other places (4.07 points in average).
School happiness	The score of upper class students (average 5.82 points) is higher compared with that of lower class students (5.13 points in average).
	The score of students living in urban areas (with the average of 5.74 points) is slightly higher in contrast to that in rural and urban areas (5.25 points).

### Scores of Three Scales for Returning College Students

**Figure 5:**
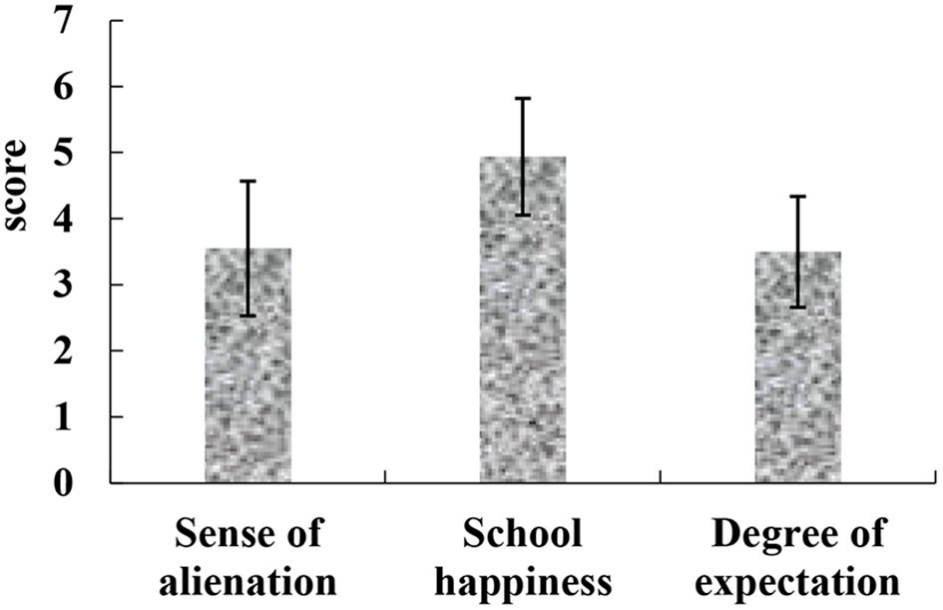
The situation of the three scales of returning college students.

After the COVID-19 pandemic, the overall degree of alienation in the physical education class of returning college students in China is relatively low, with an average score of 3.55 ± 1.018; the overall level of school happiness is relatively high, with an average score of 4.94 ± 0.883; and the overall level of expectations for a healthy life in the future is high (3.50 ± 0.840 in average). The specific results were shown in Figure 5.

### Regression Analysis

**Figure 6:**
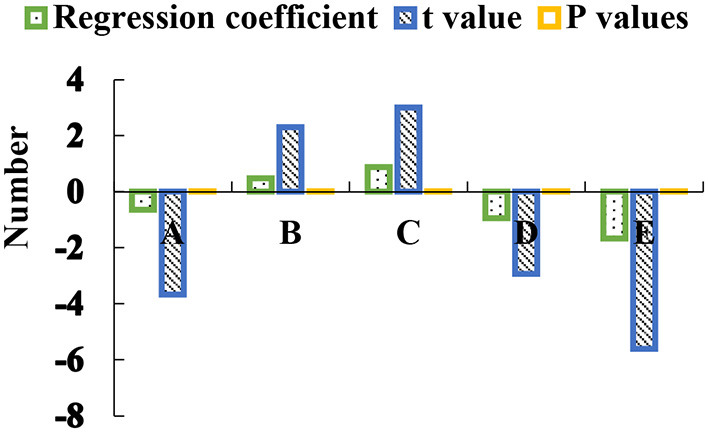
Regression analysis results of returning college students.

After the COVID-19 pandemic, the total score of college students returning to school in the alienation in physical education class is undertaken as the dependent variable and the school happiness and expectations for a healthy life in the future are taken as independent variables for multiple linear regression analysis. Stepwise regression method is used to screen out the optimal combined variable mode. The independent variables included in the model are shown in Figure 6.

In the above figure, A represents friendship happiness, B represents academic happiness, C represents freedom happiness, D represents environmental happiness, and E represents expectations for a healthy life in the future.

### Analysis of Mediation Effect

**Figure 7:**
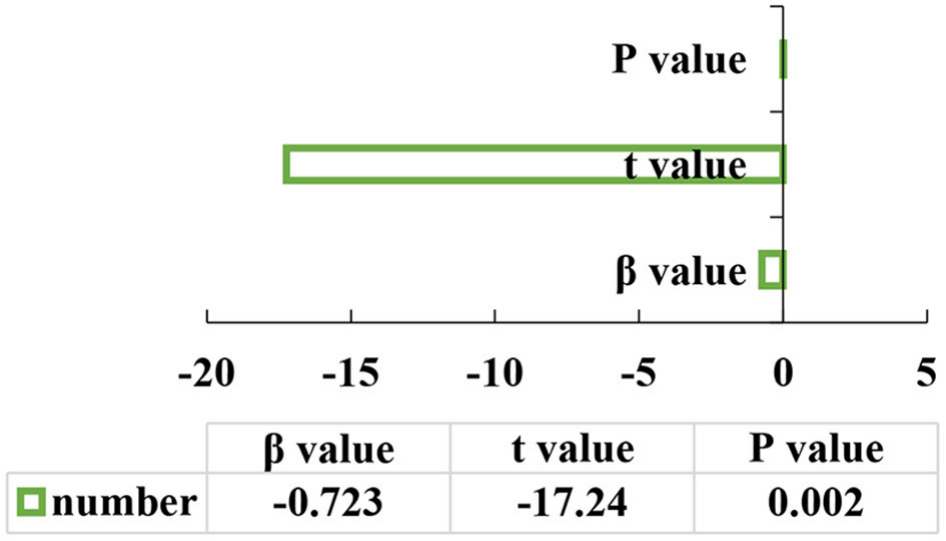
The first step of the mediation effect analysis.

**Figure 8:**
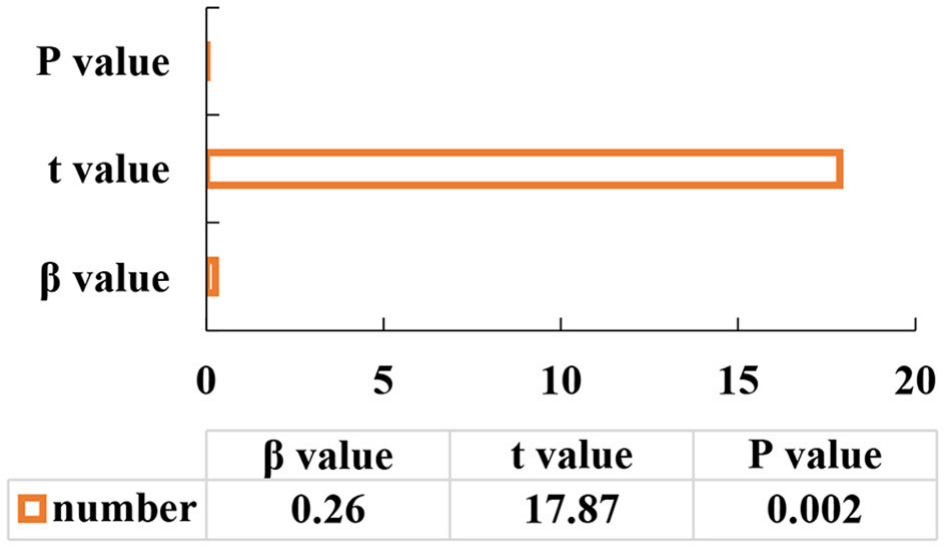
The second step of the mediation effect analysis.

**Figure 9:**
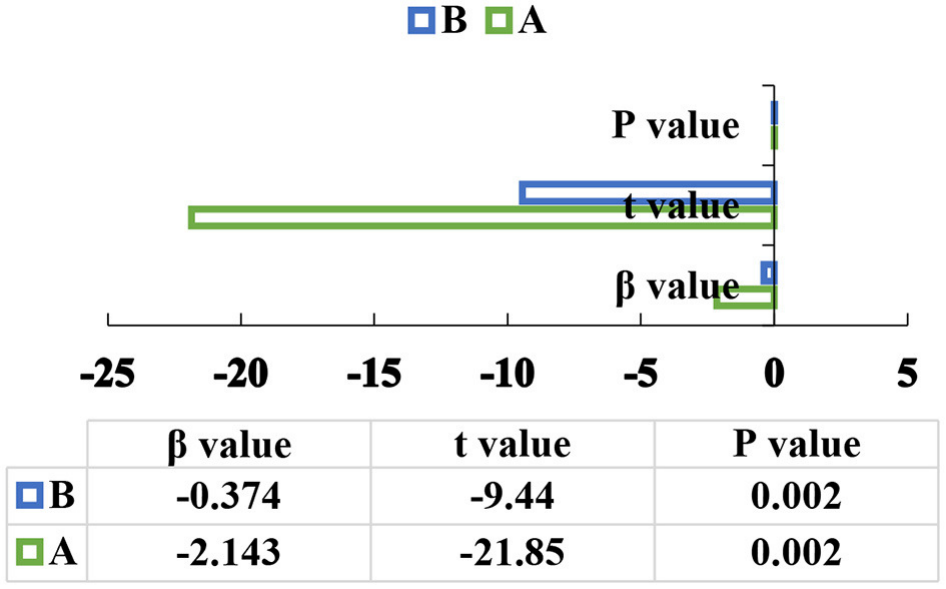
The third step of the mediation effect analysis (in the above figures, A indicates that the expectation is the independent variable, and B indicates that the school happiness is the independent variable).

The test procedure of the mediation effect should be followed. The first step: school happiness is selected as the independent variable and alienation is undertaken as the dependent variable to start the regression analysis with the equation of y = -0.723x (*P* < 0.05). The second step: the school happiness is taken as the independent variable, and the expectation degree is used as the dependent variable to start the regression analysis using m = 0.260x (*P* < 0.05). The third step: the school happiness and the expectation degree are determined as independent variables, and the alienation is used as the dependent variable for the regression analysis using y = -0.374x-2.143m (*P* < 0.05). The specific results are illustrated in Figures 7–**9**.

Figure 7 denotes that the school happiness is selected as the independent variable, and the alienation is selected as the dependent variable for regression analysis, and the *P*-value is 0.002. Figure 8 refers that the school happiness is selected as the independent variable, and expectation is the dependent variable for regression analysis, and the *P*-value is 0.002. Figure 9 indicates that the school happiness and the expectation of healthy life in the future are selected as independent variables, and alienation is used as the dependent variable for regression analysis, and the *P*-value is 0.002. It shows a positive correlation when *P* < 0.05.

## Discussion

Some experts have pointed out that COVID-19 is an infectious disease of the respiratory tract caused by coronavirus type 2 infection, which can induce severe acute respiratory syndrome. In the world, prevention and control is very difficult (31). Therefore, effective prevention and control publicity and education are needed. The results of this work show that “web browsing” and “social tools (QQ, WeChat, etc.)” are the two most important ways for returning college students to acquire knowledge about the epidemic after the COVID-19 epidemic; in addition, 73.14% of returning college students are very concerned about “the information about the epidemic reported in the media news.” The study pointed out that the most trusted source of news is the relevant information released by the official media, and the information stated by the authority has a significant inhibitory effect on the panic of the public (32). There is a positive relationship between the dissemination of information and the risk perception of COVID-19 in the official media (33). It is suggested that schools can use the “school official website, WeChat public account, and QQ group” as well as other platforms to publish publicity and education on the prevention and control of coronavirus disease.

Some documents indicate that alienation is the alienation of the relationship between the individual and the people around him and the society, the natural environment and himself. What's more, it may be controlled and dominated by the object, causing the individual to appear negative emotions such as social isolation, depression, meaninglessness of life, and self-enclosure (34). The average score of returning college students in alienation in physical education class is relatively high. This may be related to everyone's panic about the epidemic, and their lack of knowledge about the epidemic has increased the psychological pressure of returning college students and felt that life is oppressive. Such results are consistent with the findings of Masdrakis et al. (35).

In psychological research, happiness refers to an emotional state that occurs when an individual realizes that his needs are met and his ideals are realized (36). It is a complex and diverse emotional state based on the interaction between psychological factors such as needs (including motivation, desire, and interest), cognition, emotion, and external factors. Happiness indicates the assessor's assessment of the quality of life according to the set standards. The implementation of the overall assessment is a comprehensive indicator to measure the quality of life of an individual (37). In this work, the school happiness of returning college students is relatively high, perhaps because college students have good psychological quality and strong anti-stress ability, can quickly adapt to the environment, and effectively adjust their negative emotions, so that anxiety can be relieved. The average score of expectations for a healthy life in the future is also relatively high, perhaps because college students live in a relatively simple environment, and their classmates spend a long time with each other and are familiar with each other. In addition, some studies have pointed out that college students have received good higher education, have their own ideas about the future direction, and acquire knowledge relatively quickly, so they have high expectations for future life (38). The disadvantage is that the online questionnaire survey method is used, and the number of respondents is limited and there are regional differences. In future research, a systematic random sampling survey will be conducted according to different regions and different school categories to make the survey results more representative.

## Conclusions

After the COVID-19 pandemic, the cognition on the disease, alienation in physical education class, school happiness, and expectations for a healthy life in the future of Chinese returning college students are investigated and analyzed. It is found that the cognition of college students on COVID-19 is relatively poor. The overall levels of alienation in physical education classes, school happiness, and expectations for a healthy life in the future are relatively high. It can be concluded that the epidemic has a great impact on the psychology of returning college students, and it is necessary to strengthen the mental health education of college students on COVID-19. The disadvantage is that the online questionnaire survey method is used, and the number of respondents is limited and there are regional differences. In future research, a systematic random sampling survey will be conducted according to different regions and different school categories to make the survey results more representative.

## Data Availability Statement

The original contributions presented in the study are included in the article/supplementary material, further inquiries can be directed to the corresponding author/s.

## Ethics Statement

The studies involving human participants were reviewed and approved by Yangzhou University Ethics Committee. The patients/participants provided their written informed consent to participate in this study.

## Author Contributions

YX and TL: conceptualization. YT: methodology. SO: software and visualization. YT and TL: validation. YX: formal analysis, investigation, data curation, and writing—original draft preparation. KP and TL: writing—review and editing. KP and SO: supervision. All authors have read and agreed to the published version of the manuscript.

## Conflict of Interest

The authors declare that the research was conducted in the absence of any commercial or financial relationships that could be construed as a potential conflict of interest.

## Publisher's Note

All claims expressed in this article are solely those of the authors and do not necessarily represent those of their affiliated organizations, or those of the publisher, the editors and the reviewers. Any product that may be evaluated in this article, or claim that may be made by its manufacturer, is not guaranteed or endorsed by the publisher.
